# A missense mutation in the coding region of the toll-like receptor 4 gene affects milk traits in Barki sheep

**DOI:** 10.5713/ajas.19.0989

**Published:** 2020-06-24

**Authors:** Ahmed M. Sallam

**Affiliations:** 1 Animal and Poultry Production Division, Desert Research Center, Cairo 11735, Egypt

**Keywords:** Association Analysis, Milk Traits, Single Nucleotide Polymorphisms (SNP), Single Strand Conformation Polymorphism (SSCP)

## Abstract

**Objective:**

Milk production is one of the most desirable traits in livestock. Recently, the toll-like receptor (TLR) has been identified as a candidate gene for milk traits in cows. So far, there is no information concerning the contribution of this gene in milk traits in sheep. This study was designed to investigate the TLR 4 gene polymorphisms in Barki ewes in Egypt and then correlate that with milk traits in order to identify potential single nucleotide polymorphisms (SNPs) for these traits in sheep.

**Methods:**

A part of the ovine TLR 4 gene was amplified in Barki ewes, to identify the SNPs. Consequently; Barki ewes were genotyped using polymerase chain reaction-single strand conformation polymorphism protocol. These genotypes were correlated with milk traits, which were the daily milk yield (DMY), protein percentage (PP), fat percentage (FP), lactose percentage, and total solid percentage (TSP).

**Results:**

Age and parity of the ewe had a significant effect (p<0.05 or p<0.01) on DMY, FP, and TSP. The direct sequencing identified a missense mutation located in the coding sequence of the gene (rs592076818; c.1710C>A) and was predicted to change the amino acid sequence of the resulted protein (p.Asn570Lys). The association analyses suggested a significant effect (p<0.05) of the TLR genotype on the FP and PP, while the DMY tended to be influenced as well (p = 0.07). Interestingly, the presence of the G allele tended to increase the DMY (+40.5 g/d) and significantly (p<0.05 or p<0.01) decreased the FP (−1.11%), PP (−1.21%), and TSP (−7.98%).

**Conclusion:**

The results of this study suggested the toll-like receptor 4 (*TLR4*) as a candidate gene to improve milk traits in sheep worldwide, which will enhance the ability to understand the genetic architecture of genes underlying SNPs that affect such traits.

## INTRODUCTION

The dairy sheep industry plays an important role in the economics of several countries especially in the Mediterranean area. Compared to goat and cow, sheep milk has unique properties as it contains higher levels of total solids, mineral and vitamin contents [1]. Additionally, it is more nutritious, richer in vitamins A, B, and E, calcium, phosphorus, potassium and magnesium, and has a health benefit as it contains a higher portion of short and medium fatty acids chains [2]. On the other hand, it has a positive impact on the newborn lambs, in which good milk supply from the mother may increase the growth rate in the lambs [3].

**Figure 1:**
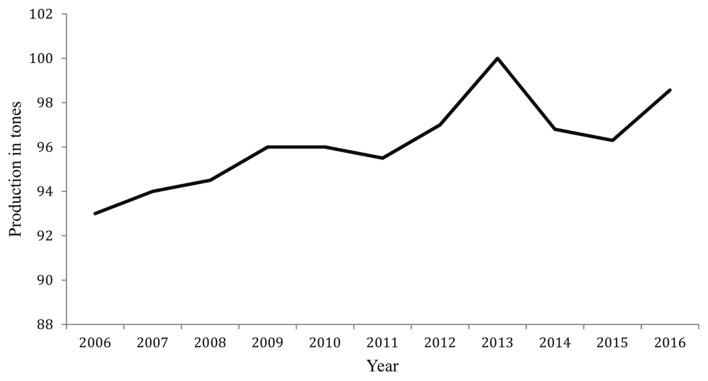
Total production of sheep milk in the last ten years in Egypt retrieved from FAO statistics database (http://www.fao.org/faostat/en/#home).

The Egyptian dairy production industry in predominantly based on cows and buffaloes, with little concern for sheep and goats. This is may be due to [4] the management system of the milk-producing animals in Egypt, which is often the subsistence and smallholder systems. However, sheep contribute about 98,570 tonnes of milk, which is about 5% of the total whole milk produced annually in Egypt with a reasonable increase in the total milk production from sheep in the last few years (Figure 1; http://www.fao.org/faostat/en/#data). Moreover, sheep milk is preferable for the Bedouin and contributes a substantial part of their livelihood. Unfortunately, there are negative perspectives concerning the small potential for milk production of the Egyptian sheep, which influences their inclusion in the breeding objectives and selection programs. In Egypt, Barki sheep are considered as one of the three major sheep breeds [5]. They are dominate in the North Western Coastal Zone, extending from the Libyan boarders to the west of Alexandria of Egypt with a total of population near to 406,360 [6], which is estimated to be about 8% of the total number of sheep population in Egypt (http://www.fao.org/faostat/en/#data). Barki sheep are well adapted to live and produce under hard conditions of shortage food supply and water resources.

Recently, several genotyping technologies of single nucleotide polymorphisms (SNPs) have been used to identify markers associated with specific phenotypes [7,8]. Milk production traits are important targets for genetic improvement and early predictions using genetic markers are therefore important criteria in livestock breeding. Genetic polymorphisms in several genes associated with milk traits in livestock have been reported. The importance of these variants is due to their effect on milk production, composition and quality. Consequently, identifying genetic markers that are associated with the desired phenotype could be helpful in selection programs in livestock [8].

The toll-like receptor 4 (TLR4) is one of the TLRs families, which are known as pathogen-recognizing molecules. Recently, a number of studies reported the association between TLR4 polymorphisms and lactation persistency in Canadian dairy cattle [9], percentages of fat and protein in milk in Irish cattle [10], somatic cell score and 305-day milk yield in Iranian Holstein cattle [11], and milk yield and fat percentage in Holstein cows in New Zealand [12]. Nevertheless, these associations have neither reported nor studied in sheep.

The ovine *TLR4* gene is located on chromosome 21 and consists of 9,069 base pairs and 9 exons and introns. Information regarding the genetics of sheep milk is scarce in the Egyptian breeds. The objective of this study was to identify genetic variants potentially associated with milk traits in Barki sheep, which may be used to make predictions about the superior ewes kept for dairying, selection programs and genetic improvement strategies in sheep in Egypt and worldwide.

## MATERIALS AND METHODS

### Ethics statement

All procedures were conducted and approved by the Animal Care and Use Committee of Desert Research Center, Egypt, and comply with the guidelines and regulations of the Animal Ethics Committee Institute of the European Parliament for protection of experimental animals (2010/63/EU).

### Animal resources and phenotypes

This work was carried out at the facilities of Maryout and Matrouh Research Stations and the Animal Molecular Genetics Laboratory, which both belong to the Animal and Poultry Production Division at Desert Research Center (DRC). Approximately 311 milk-producing ewes were recorded during the season of 2017 through 2018 for the following milk traits: daily milk yield (DMY), total fat percent (FP), total protein percent (PP), lactose percent (LP), and total solid percent (TSP). The milk composition was analyzed using a MilkoScan (130 A/SN. Foss Electric, Hilleroed, Denmark).

The test-day protocol was considered to collect milk records. The milk yield was recorded biweekly from the time of parturition till the 12th week using hand-milking technique. Lambs were separated from their dams 12 h before milking. Then, right half of the udder was milked in the morning by hand while the lambs were allowed to suckle from the left half. Another milking was carried out in the evening by the same way starting with the left udder. Milk yield per day was measured accordingly in milliliter by summation the morning and evening milking multiplying by two. Of the 311 ewes originally were available for blood collection and genotyping, the phenotypic records were available for about 256 ewes, which were milking producing ewes, and were included in the subsequent association analyses.

### Genetic analysis

#### Selection of the candidate gene

The TLR4 is a newly identified candidate gene for milk production traits in Holstein cattle in New Zealand [12] and other cattle breeds worldwide. In sheep, the gene has not been characterized or investigated for its contribution to milk traits yet. Based on protein sequence of *Ovis aries* of the selected gene, the following similarities were obtained by comparing of the amino acid sequence of the gene with three other animal species (Table 1).

#### Genotyping and identifying the polymorphisms

Genomic DNA was extracted from the whole blood samples using Intron bio (commercial kits, Germany) following the manufactures protocol. Finally DNA samples were stored in a laboratory freezer at −20°C. A part of the *Ovis aries TLR 4* gene (GeneBank: DQ922636.1) was amplified by polymerase chain reaction (PCR) using the following specific forward and reverse primers: F: 5-^1309^TCAGGTGCTGAATATGAG TCA^1309^-3′ and R: 5-^1742^CTCTCACCCCTGCCATACTT^1761^-3′ (https://www.ncbi.nlm.nih.gov/gene/?term=DQ922636.1). The PCR products (453 bp) were amplified using thermal cycler apparatuses in tubes containing a 12.5 μL of PCR mixture containing 0.8 U Taq DNA polymerase (Qiagen, Hilden, Germany). To amplify the target region, the PCR conditions were as follows; initial step of one cycle of 5 min. at 95°C, followed by 35 cycles of 1 min at 95°C, 1 min at 58°C, 1 min 30 s at 72°C with final extension of 10 min at 72°C. The amplified regions were detected and confirmed using agarose gel electrophoresis.

PCR-single strand conformation polymorphism (SSCP) technique was used to genotype the ovine TLR4 in Barki ewes. Briefly, a 15-μL aliquot of each amplicon was denatured at 95°C for 5 min, the samples were placed in wet ice and immediately loaded onto 12% acrylamide: bisacrylamide (37.5:1; Bio-Rad, Hercules, CA, USA) gels. Electrophoresis for 18 h in 0.5× Tris/borate/ethylenediaminetetraacetic acid (TBE) buffer at 200 V was undertaken in Bio-Rad Protean II xi cells with water circulation at 25°C. The gels were silver-stained using the method of Zhou et al [12].

### DNA sequencing

To identify the polymorphic SNPs, PCR amplicons representing different SSCP banding patterns were delivered to Macrogen sequencing company (Seoul, Korea) for sequencing in both directions according to the BigDye terminator protocol. DNA sequences for the forward primers were aligned against the reference sequences from NCBIdb for the corresponding amplified region (ie. flanking region, exon). The alignment was also conducted against the sequence of the reverse primer of the corresponding region to ensure that the identified SNP is real and not artifact. Identification of SNPs was performed using 4Peaks software for the pairwise alignment. Subsequently, the screened polymorphic SNPs were compared to the available SNP NCBI database to determine their exact locations in the gene (https://www.ncbi.nlm.nih.gov/gene/?term=DQ922636.1).

### Association analysis

The association analyses between the ovine TLR4 genotypes and milk production traits was performed using the general linear model process in SAS using the following liner model:

$${\text{Y}}_{\text{ijklm}}=\mu +{\text{G}}_{\text{i}}+{\text{Y}}_{\text{sj}}+{\text{H}}_{\text{k}}+{\text{e}}_{\text{ijklm}},$$

Where, Y_ijklm_ = the trait of interest; μ = the overall mean; G_i_ = the effect of genotype; Y_sj_ = the effect of parity of the ewe (5 parities); H_k_ = the effect of age of the ewe (5 level: 1st level, animals ≤3 years old; 2nd level, animals at 4 to 5 years old; 3rd level, animals at 6 to 7 years old; 4th level, animals at 8 years old; and 5th level, animals above 8 years old); and e_ijklm_ = random error. The age of dam and parity were included in the model, while the type of birth (singles or twinning) was not considered, as all births were singles.

Additionally, the additive (A) and dominant (D) effects of each genotype were tested. Accordingly, the genotype effect was divided into A and D effects as follow:

$$\begin{array}{l} \text{The additive effect }(\text{A})=\frac{\text{GG}-\text{TT}}{2}\\ \text{The dominant effect }(\text{D})=\text{GT}-\frac{\text{GG}+\text{TT}}{2} \end{array}$$

Where, GG, GT, and TT represent the least square means of the corresponding genotypes, respectively.

## RESULTS

### Descriptive statistics and selection of the candidate gene

Results of protein sequence similarity in the three species suggested a high similarity of the gene (Table 1), which means that this gene may be a good target to be investigated in sheep. The milk production traits for Barki ewes included in the current study are described in Table 2. The DMY ranged from 0.07 to 0.88 kg/d with an average of 0.31±0.13 kg/d. The FP ranged from 1.45% to 11% with an average of 4.63%±0.15%. The PP ranged from 1.75% to 10% with an average of 5.51% ±0.14%. The LP ranged from 1.02% to 10% with an average of 6.37%±0.16%. The TSP ranged from 2.55% to 35% with an average of 17.37%±0.66%.

### Effect of the age of ewe and parity on the milk traits

**Figure 2:**
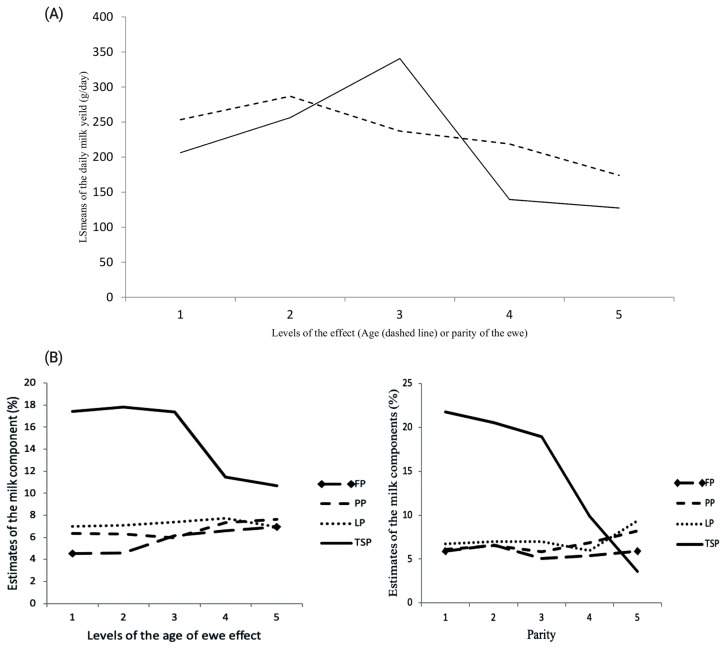
(A) Effect of the age of ewe and parity (5 parities) on the daily milk yield, in which age of the ewe had 5 levels: 1st level, animals ≤3 years old; 2nd level, animals at 4 to 5 years old; 3rd level, animals at 6 to 7 years old; 4th level, animals at 8 years old; 5th level, animals above 8 years old. (B) Effect of the age of ewes and parity on milk compositions. FP, fat percentage; PP, protein percentage; LP, lactose percentage; and TSP, total solids percentage. The age and parity of the ewe significantly affected (p<0.05 or p<0.01) daily milk yield, FP and TSP.

The age of ewe and parity significantly influenced the DMY, FP, and TSP (Table 3, Figure 2). Ewes at 3 and 4 years old tended to produce the highest DMY 286.87±31.37 g/d followed by ewes at 5 and 6, 253.47±41.76 g/d, while the DMY was the lowest from ewes above 8 years old (174.01±82.68 g/d). Similarly, the TSP tended to decrease in milk as the ewe aged, as it was 17.81%±1.46%, 17.43%±1.95%, and 10.66%± 3.53%, for the corresponding levels of age, respectively. Conversely, the FP in milk was the lowest at ages 3 and 4 years old (4.53%±0.55%) followed by 5 and 6 years old ewes (4.59% ±0.41%) while it was the highest at the above 8 years old ewes (6.92%±0.99%).

Likewise, the effect of parity of ewe on milk traits was highly significant (p<0.01). Ewes at third parity tended to produce the highest DMY (340.62±32.46 g/d) followed by ewes at the second parity (256.33±34.31 g/d), while the ewes at the fifth parity produced the lowest DMY (127.36±94.21 g/d). Similarly, ewes in the first parity produced the highest TSP in milk (21.78%±2.11%) compared to those who are in the fifth parity, which produced the lowest TSP (3.60%±4.05%). Conversely, ewes in the second parity produced the highest FP in milk (6.63%±0.45%) and ewes at the third parity were the lowest FP producers (5.04%±0.43%).

Nevertheless, both factors did not significantly (p<0.05) affect the percentages of protein (PP) and lactose (LP) in the milk of the Barki ewes.

### The toll-like receptor 4 variation in Barki ewes

**Figure 3:**
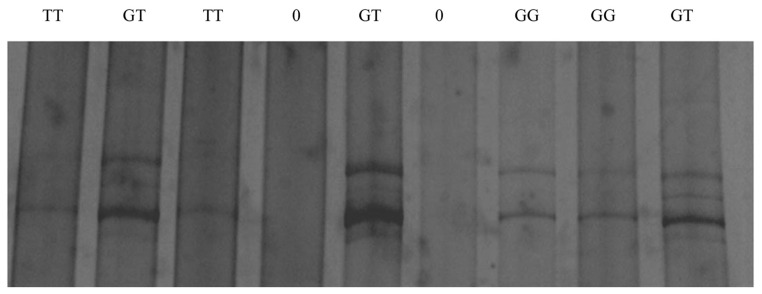
PCR-SSCP patterns of the ovine *TLR4* gene in Barki sheep representing G and T alleles and different genotypes. PCR, polymerase chain reaction; SSCP, single strand conformation polymorphism; *TLR4*, toll-like receptor 4.

**Figure 4:**
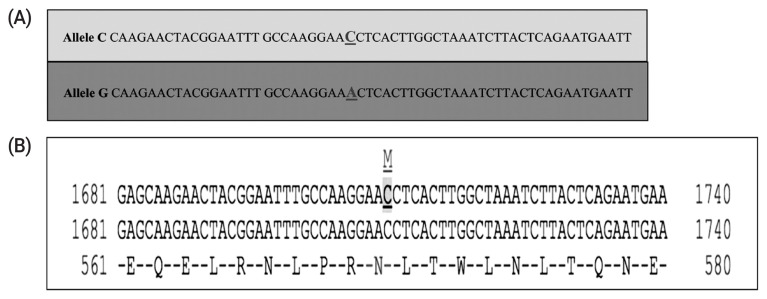
(A) Sequencing results of the ovine *TLR4* variants showing the identified SNPs and their locations. (B) The predicted consequences of the identified variant of the ovine *TLR4* gene on the resulted protein (ENSOARP00000006210.1: p.Asn570Lys) in Barki sheep from Ensembl (www.ensembl.org). *TLR4*, toll-like receptor 4; SNPs, single nucleotide polymorphisms.

The PCR-SSCP patterns identified two distinctive conformation patterns (named G and T) in the TLR4 in the investigated ewes (Figure 3). The allelic frequencies were 63.5% and 36.5% for G and T alleles, respectively. Likewise, the genotypic frequencies were 44.6%, 35.7%, and 19.7% for GG, GT, and TT genotypes, respectively. DNA sequencing results of the representative patterns identified a missense mutation located in the coding sequence of exon 3 of the gene at position 5858783 base pairs of chromosome 2 (ENSOART00000006304.1: c.1710C>A). Interestingly, the identified SNP (rs592076818) was predicted to change in the amino acid sequence (Figure 4) of the resulted protein (ENSOARP00000006210.1: p.Asn570Lys; https://www.ensembl.org/Ovis_aries/Transcript/).

### Effect of variants in toll-like receptor 4 gene on milk traits

Results of the presence/absence allele model clearly showed that (Table 4) the presence of the G allele increased the DMY (presence: 310.36±14.12 g/d, absence: 269.86±36.14 g/d), however, this effect was not significant (p = 0.17). Moreover, the FP (presence, 4.53±0.15; absence, 5.64±0.16), PP (presence, 5.40±0.15; absence, 6.61±0.56) decreased significantly (p< 0.05), and TSP (presence, 16.52±0.70; absence, 24.50±1.6) with the presence of the G allele. Nonetheless, this model did not show any significant with the T allele.

### Effect of variations in toll-like receptor 4 gene on milk traits

Results of the association analyses of the TLR4 genotypes and milk traits (Table 5) suggested that ewes with the GG genotype significantly produced more milk (DMY = 246.42±31.07; p = 0.07) with lower FP (5.35±0.39; p = 0.024) and PP (5.90± 0.39; p = 0.020). In contrary, ewes with the TT genotype significantly produced higher FP (6.88±0.63), PP (7.80±0.63), and lower milk production (DMY = 141.96±44.47). Nonetheless, TLR4 genotypes did not significantly affect the LP and TSP in Barki ewes’ milk.

The genetic effects (additive/dominance) of the TLR4 variants on the studied milk traits in Barki sheep are presented in Table 6. The results showed significant additive effects on the FP (−0.72, p<0.01), PP (−0.86, p<0.01), LP (−0.49, p = 0.05), and TSP (−2.73, p<0.01). Furthermore, a significant dominance effect was also observed for the TLR4 variants on the PP (+0.68, p<0.05).

## DISCUSSION

Despite the reported high heritability of milk traits in livestock, which suggests that selection for these traits could be made based on the traditional methods, identifying genetic markers and candidate genes to improve milk traits will assist selecting the superior animals early in their ages with more success [13]. On the other hand, although a number of studies have investigated variations in TLR4 in bovine and its relationship with milk traits, little has been undertaken with this gene in sheep. Accordingly, critical investigations are required to identify the gene variations in sheep and to estimate their effects on milk. This study describes for the first time genetic variants within a part of the ovine *TLR4* gene and their association with important milk production traits in Barki ewes.

Overall, the estimates of milk traits reported here in Barki ewes were in agreement with those estimated by Abousoliman et al [3] in Barki sheep and dairy sheep in Slovakia [14]. However, some milk components were dissimilar in the Slovakian ewes (FP = 6.91% vs 4.63%, LP = 4.54% vs 6.37%) compared with those reported in this study. Likewise, Ozmen and Kul [15] reported different estimates for the FP, PP, and LP in Sakis (7.06, 6.43, and 5.41, respectively), Akkaraman (4.12, 4.02, and 6.11, respectively) and Awassi (5.17, 3.91, and 5.92, respectively) sheep breeds, than those reported here in Barki ewes. Nevertheless, these values were slightly higher than those reported by Balthazar et al [16] in sheep, goat and cows, and in the dairy ewes in the US [17].

Consistent with the results of the current study, the age of ewe and parity significantly (p<0.05) affected most of the milk production traits and composition in cattle [18], goats [19], and sheep [17,20–22] in different breeds worldwide. Results of the current study showed similar trends of both effects (i.e. age and parity) on milk yield and composition in Barki ewes, in which the younger ewes tended to produce more DMY and TSP and lower FF, PP, and LP compared to older ewes. These findings confirmed the negative correlation between the milk yield and components at different ages and parities. However, a non significant effect of the age of ewe on DMY was reported in Rahmani and Chios breeds in Egypt [20]. According to the results of this study, selection for ewes at 4 years old and the third parity could increase the daily milk production as well.

The only identified SNP in the investigated area of the TLR4 was lower than the number of SNPs identified in the same corresponding region of the gene in sheep and goats, which were 14 [23] and 4 [24], respectively. This suggests a low genetic diversity in the gene in Barki sheep, which may be because of the high selection pressure for this gene in Barki sheep [12]. This result disagrees with earlier reports that stated that the immune response genes in ovine have a high level of diversity compared to their orthologous genes in bovines [25]. However, only one SNP has been identified (c.2021C/T; rs8193069) in the coding region of the bovine TLR4 [12]. Moreover, the missense mutation identified here supports the findings of Zhou et al [23] that reported most of the SNPs in this region of the ovine TLR4 were non-synonymous causing amino acid changes and creating a polymorphic protein. A possible reason for that is to guarantee a large amount of diversity in a gene required for disease resistance.

Notably, the identified SNP is located at the 1,710 position of the coding region of the ovine TLR4 region (c.1710C>A), which changes the Asparagine amino acid at position 570 to the lysine in the resulted protein (p.Asn570Lys). Results of the association analysis of the TLR4 genotypes and milk traits support our hypothesis that there is genetic potential for improvement in milk production in Barki sheep. Genetic variations in milk production and composition were clearly observed, which emphasises the importance of finding genetic makers for the marker-assisted selection programs in the breed. So far, there is no study that has investigated the association between TLR4 variants and milk traits in sheep. So that, it is hard to compare the results of the current study taking into account the perspectives of other researchers’ findings.

Noteworthy, the association tests showed that the GG genotype positively affected DMY (246.42±31.07 g/d) but negatively affected FP (5.35%±0.39%) and PP (5.90%±0.39%) compared to the TT genotype (141.96±44.47, 6.88±0.63, and 7.80±0.63, for the corresponding traits, respectively). This means that animals that carry the G allele produce more daily milk with lower fat and protein percentages, which also supports the previously negative genetic correlation between DMY and milk compositions [26]. These results were compatible with the results of the presence/absence allele model, which clearly showed that the presence of the G allele increased the DMY (presence, 310.36±14.12 g/d; absence, 269.86±36.14 g/d), however, this effect was not significant (p = 0.17), and decreased the FP (presence, 4.53±0.15; absence, 5.64±0.16) and PP (presence, 5.40±0.15; absence, 6.61±0.56).

Importantly, it was suggested that the separation of the genetic variants of a milk-candidate gene into a good and a poor genotypes in relation to milk traits will ease the future breeding towards better milk production and composition [27]. This suggestion could be implied here in the TLR4 variants to achieve the optimum progress in dairy sheep products based on the results of the current report. Recent reports refer to the importance of estimating the additive and/or the dominance genetic effect of specific variants on the economic traits of livestock [28,29]. The significant additive effects of the TLR4 variants identified here in Barki ewes on FP, PP, LP, and TSP suggested that these effects should be considered in genetic improvement of milk production in Barki sheep. Moreover, the considerable additive and dominance effects that were observed here for the studied variant on the PP emphasizing the significant of the association and increase the accuracy of the potential prediction of the animal [28]. Similar significant effects of the non-additive genetic models were reported for milk traits in cattle [30].

There is speculation that of the low frequency of the SNP c. 2012C/T in the TLR4 gene in the Canadian Holstein [31] is a consequence of natural selection against the allele, as it may cause an immature immune response. This may be a reason for the low frequency of the T allele in Barki sheep as well. Overall, the biological mechanism behind the effect of the TLR 4 on milk traits is still unknown and needs intensive investigations to be understood. However, it could be attributable to the contribution of the gene in specific signaling pathways that influences the expression of major gens for milk traits, which modulates the milk traits as an end product [32]. Another possible explanation is the demonstrated correlation between the TLR polymorphisms and clinical diseases (i.e. mastitis and Johne’s disease) in livestock that negatively affect milk quantity and quality [9,31]. Thus, the identified TLR4 variants identified here may positively affect milk components by improving the immune response of the animal against the previously mentioned milk diseases. It is important to understand the reasons behind the low frequency of the allele T in Barki sheep, further investigations for the association of sheep that have the c.1710C>A mutation in context of the immune responses are highly recommended.

Summarizing, this report in presents different perspectives concerning the milk production potentials in the indigenous Egyptian sheep. It confirms the relationship between the *TLR4* gene and milk traits in livestock as suggested by several reports, and in sheep for the first time. Importantly, the additive/dominance modes reported here enables understanding of the inheritance modes of the TLR4 markers in milk traits of sheep in general and Barki in particular. Accordingly, this information may be used to identify the superior animals for successful genetic improvement strategies.

## Figures and Tables

**Table 1:** Comparative analysis of amino acid sequences of the selected genes in different species

Gene	Organism	Accession number	Similarity1) (%)
*TLR* (*Ovis aries*) (NP_001108139.1)	Arabian camel	XP_010977688.1	86
	Buffalo	XP_006068111.1	96
	*Bos taurus*	NP_776739.1	93

1) The similarity alignment was based on *Ovis aries* (sheep) published on NCBI/BLAST.

**Table 2:** Descriptive statistics of the milk production traits in Barki ewes

Item	Average	SE	Minimum	Maximum
DMY (kg/d)	0.31	0.13	0.07	0.88
FP	4.63	0.15	1.45	11
PP	5.51	0.14	1.75	10
LP	6.37	0.16	1.02	10
TSP	17.27	0.66	2.55	35

n = 233 ewes.

SE, standard error; DMY, daily milk yield (g/d); FP, fat percentage; PP, protein percentage; LP, lactose percentage; TSP, total solid percentage.

**Table 3:** Effect of the non-genetic factors on milk traits in Barki ewes

Factor1)	n	Traits				

		DMY	FP	PP	LP	TSP
Age of ewe						
1st	17	253.47±41.76	4.53±0.55	6.35±0.54	6.97±0.65	17.43±1.95
2nd	48	286.87±31.37	4.59±0.41	6.29±0.41	7.08±0.48	17.81±1.46
3rd	13	237.12±40.9	6.18±0.54	5.97±0.54	7.37±0.64	17.40±1.93
4th	14	218.77±46.22	6.61±0.59	7.34±0.59	7.75±0.70	11.47±2.11
5th	4	174.01±82.68	6.92±0.99	7.63±0.99	6.96±1.17	10.66±3.53
Significance		0.031*	0.001^**^	0.15	0.87	0.039*
Ewe parity						
1st	14	206.38±43.03	5.87±0.59	6.11±0.59	6.73±0.70	21.78±2.11
2nd	32	256.33±34.31	6.63±0.45	6.54±0.45	6.99±0.53	20.58±1.61
3rd	30	340.62±32.46	5.04±0.43	5.83±0.42	7.0±0.50	18.93±1.52
4th	17	139.53±41.15	5.40±0.50	6.89±0.50	5.99±0.60	9.89±1.80
5th	3	127.36±94.21	5.90±1.14	8.20±1.14	9.37±1.35	3.60±4.05
Significance		0.001^**^	0.035*	0.28	0.17	0.0001^**^

n = 233 ewes; predicted least square means±standard errors from general linear models.

DMY, daily milk yield (g/d); FP, fat percentage; PP, protein percentage; LP, lactose percentage; TSP, total solid percentage.

1) Levels of age of the ewe: 1st level, animals ≤3 years old; 2nd level, animals at 4 to 5 years old; 3rd level, animals at 6 to 7 years old; 4th level, animals at 8 years old; 5th level, animals above 8 years old.

* Significance estimated at p<0.05 and p<0.01 for the highly significant.

**Table 4:** Association of presence or absence of the toll-like receptor variants with milk production traits in Barki ewes

Trait	Variant	Presence	Absence	Effect1)	p-value
	
		Mean±SE2)	Mean±SE2)		
DMY	T	306.51±22.02	307.05±14.99	−1.46	0.31
	G	310.36±14.12	269.86±36.14	+40.5	0.17
FP	T	5.06±0.26	4.20±0.16	+0.86	0.82
	G	4.53±0.15	5.64±0.16	−1.11	0.022*
PP	T	6.03±0.24	4.98±0.13	+1.05	0.10
	G	5.40±0.15	6.61±0.56	−1.21	0.007**
LP	T	6.66±0.26	6.07±0.18	+0.59	0.56
	G	6.30±5.95	7.07±6.06	−0.77	0.17
TSP	T	18.23±1.11	16.22±0.7	+2.01	0.94
	G	16.52±16.15	24.50±20.98	−7.98	0.017*

SE, standard errors; DMY, daily milk yield (g/d); FP, fat percentage; PP, protein percentage; LP, lactose percentage; TSP, total solid percentage.

1) Increase (+) or decrease (-) of the corresponding trait.

2) Predicted least square means±SE from general linear models.

Significance estimated at

* p<0.05,

** p<0.01.

**Table 5:** Effect of the toll-like receptor genotypes on milk production traits and composition in Barki ewes

Trait	Genotype (n)			p-value

	TT (47)	GT (85)	GG (106)	
DMY (g/d)	141.96±44.47	233.76±28.10	246.42±31.07	0.07
FP	6.88±0.63	5.07±0.34	5.35±0.39	0.024*
PP	7.80±0.63	6.45±0.34	5.90±0.39	0.020*
LP	7.43±0.75	7.09±0.41	7.16±0.42	0.89
TSP	17.25±2.25	13.92±1.24	13.69±1.41	0.28

n = 233 ewes.

DMY, daily milk yield (g/d); FP, fat percentage; PP, protein percentage; LP, lactose percentage; TSP, total solids percentage.

* Significance estimated at p<0.05.

**Table 6:** Genetic effect of the ovine toll-like receptor 4 on milk traits in Barki ewes

Traits	Genetic effect1)			

	Additive	p-value	Dominance	p-value
DMY (g/d)	8.10	0.69	12.90	0.63
FP	−0.72	0.003**	0.52	0.10
PP	−0.86	<0.001**	0.68	0.02*
LP	−0.49	0.05*	0.34	0.30
TSP	−2.73	0.007**	−0.57	0.67

DMY, daily milk yield (g/d); FP, fat percentage; PP, protein percentage; LP, lactose percentage; TSP, total solid percentage.

1) Increase (+) or decrease (−) of the corresponding trait.

Significance estimated at

* p<0.05,

** p<0.01.
